# Influence of POR*28 Polymorphisms on CYP3A5*3-Associated Variations in Tacrolimus Blood Levels at an Early Stage after Liver Transplantation

**DOI:** 10.3390/ijms21072287

**Published:** 2020-03-26

**Authors:** Takahiro Nakamura, Mio Fukuda, Ryosuke Matsukane, Kimitaka Suetsugu, Noboru Harada, Tomoharu Yoshizumi, Nobuaki Egashira, Masaki Mori, Satohiro Masuda

**Affiliations:** 1 Department of Clinical Pharmacology and Biopharmaceutics, The Pharmaceutical College, Kyushu University, 3-1-1 Maidashi, Higashi-ku, Fukuoka 812-8582, Japan; nakamura.takahiro.551@s.kyushu-u.ac.jp; 2 Department of Pharmacy, Kyushu University Hospital, 3-1-1 Maidashi, Higashi-ku, Fukuoka 812-8582, Japan; miomio15@pharm.med.kyushu-u.ac.jp (M.F.); mryosuke@pharm.med.kyushu-u.ac.jp (R.M.); suetsugu@pharm.med.kyushu-u.ac.jp (K.S.); n-egashi@pharm.med.kyushu-u.ac.jp (N.E.); 3 Department of Surgery and Science, Graduate School of Medical Sciences, Kyushu University, 3-1-1 Maidashi, Higashi-ku, Fukuoka 812-8582, Japan; nharada@surg2.med.kyushu-u.ac.jp (N.H.); tomyoshi@surg2.med.kyushu-u.ac.jp (T.Y.); m_mori@surg2.med.kyushu-u.ac.jp (M.M.); 4 Department of Pharmacy, International University of Health and Welfare Narita Hospital, 852 Hatakeda, Narita 286-0124, Japan

**Keywords:** tacrolimus, CYP3A5*3, POR*28, liver transplantation, biomarker

## Abstract

It is well known that the CYP3A5*3 polymorphism is an important marker that correlates with the tacrolimus dose requirement after organ transplantation. Recently, it has been revealed that the POR*28 polymorphism affects the pharmacokinetics of tacrolimus in renal transplant patients. In this study, we examined whether POR*28 as well as CYP3A5*3 polymorphism in Japanese recipients and donors would be another biomarker for the variation of tacrolimus blood levels in the recipients during the first month after living-donor liver transplantation. We enrolled 65 patients treated with tacrolimus, who underwent liver transplantation between July 2016 and January 2019. Genomic DNA was extracted from whole-blood samples, and genotyping was performed to examine the presence of CYP3A5*3 and POR*28 polymorphisms in the recipients and donors. The CYP3A5*3/*3 genotype (defective CYP3A5) of the recipient (standard partial regression coefficient [median C/D ratio of *CYP3A5* expressor vs. *CYP3A5* non-expressor, *p* value]: Pod 1–7, β= −0.389 [1.76 vs. 2.73, *p* < 0.001]; Pod 8–14, β = −0.345 [2.03 vs. 2.83, *p* < 0.001]; Pod 15–21, β= −0.417 [1.75 vs. 2.94, *p* < 0.001]; Pod 22–28, β = −0.627 [1.55 vs. 2.90, *p* < 0.001]) rather than donor (Pod 1–7, β = n/a [1.88 vs. 2.76]; Pod 8–14, β = n/a [1.99 vs. 2.93]; Pod 15–21, β = −0.175 [1.91 vs. 2.94, *p* = 0.004]; Pod 22–28, β = n/a [1.61 vs. 2.67]) significantly contributed to the increase in the concentration/dose (C/D) ratio of tacrolimus for at least one month after surgery. We found that the tacrolimus C/D ratio significantly decreased from the third week after transplantation when the recipient carried both CYP3A5*1 (functional CYP3A5) and POR*28 (*n* = 19 [29.2%], median C/D ratio [inter quartile range] = 1.58 [1.39–2.17]), compared with that in the recipients carrying CYP3A5*1 and POR*1/*1 (*n* = 8 [12.3%], median C/D ratio [inter quartile range] = 2.23 [2.05–3.06]) (*p* < 0.001). In conclusion, to our knowledge, this is the first report suggesting that the POR*28 polymorphism is another biomarker for the tacrolimus oral dosage after liver transplantation in patients carrying CYP3A5*1 rather than CYP3A5*3/*3.

## 1. Introduction

Tacrolimus (TAC) is a calcineurin inhibitor widely used to prevent graft rejection after organ transplantation. It is essential to maintain the target TAC trough concentrations through therapeutic drug monitoring (TDM), as individual variations in the pharmacokinetics of TAC are large, and the therapeutic window is narrow [1,2]. Some patients experience TAC variations above or below the therapeutic range, despite TDM, and are at risk of renal toxicity or acute cellular rejection [3,4].

It is well known that TAC is metabolized by cytochrome P450 3A (CYP3A), and single-nucleotide polymorphisms in the *CYP3A5* gene contribute to the pharmacokinetic variability of TAC [5,6]. In particular, many studies have shown that homozygous carriers of the CYP3A5*3 allele have a higher dose-normalized trough concentration of TAC than do CYP3A5*1 carriers [7,8,9,10,11,12,13]. The CYP3A5*3 allele is a mutation in the intron region that causes alternative splicing, resulting in a truncated protein and a severe decrease of a functional CYP3A5 enzyme [14,15]. Patients with the homozygous CYP3A5*3 genotype (CYP3A5*3/*3) have no CYP3A5 activity and show a significantly higher concentration/dose (C/D) ratio of TAC than CYP3A5*1 carriers.

Cytochrome P450 oxidoreductase (POR), which transfers electrons from NADPH to a CYP enzyme, is expressed in a wide range of tissues and plays an important role in the CYP-mediated drug oxidation process [16]. Gene mutations in *POR* range from rare ones that cause bone malformations, steroid synthesis disorders, and genital lesions [17,18] to more common ones, with a high frequency of expression. Among the latter, POR*28 is considered to be one of the most frequent and best-studied polymorphisms. POR*28 leads to an amino acid substitution (A503V), which affects the flavin adenine dinucleotide-binding site of POR and is believed to alter its reactivity toward CYP enzymes [19,20]. Recently, several reports have demonstrated, mainly in renal transplant recipients, that the POR*28 polymorphism enhances TAC metabolism through excessive activation of CYP3A5 [21,22,23,24,25]. Some reports have shown that POR*28 (T-allele) carriers had significantly lower TAC C/D ratio than non-carriers in CYP3A5 expressors but not in CYP3A5 defective [21,24], whereas others have shown that the POR*28 polymorphism reduces the TAC C/D ratio regardless of the presence or absence of *CYP3A5* expression [22,23]. However, to our knowledge, there have been no reports that evaluated the effects of the POR*28 polymorphism in liver transplant patients.

The purpose of this study was to investigate the influence of POR*28 on CYP3A5*3-associated variations in the TAC C/D ratio during the first month following living-donor liver transplantation.

## 2. Results

### 2.1. Patients’ Characteristics

The characteristics of the recipients and donors from this study are shown in Table 1. Among preoperative clinical laboratory values, high levels of aspartate aminotransferase and total bilirubin and low levels of serum albumin were observed, but there were no other remarkable test results. Among primary diseases, alcoholic liver disease was the most common hepatocellular disease, and primary bile cholangitis was the most common cholestatic disease. All patients were 18 years of age or older. The median age of the recipients was 59 years, and that of the donors was 39 years. There were 28 male and 37 female recipients, while 43 donors were males and 22 were females.

### 2.2. Demographics of Genetic Polymorphisms

The allele frequencies of *CYP3A5* were examined in the present study as follows: recipients: *1/*1, *n* = 2 [3.1%]; *1/*3, *n* = 25 [38.5%]; *3/*3, *n* = 38 [58.5%]; donors: *1/*1, *n* = 3 [4.6%]; *1/*3, *n* = 27 [41.5%]; *3/*3, *n* = 35 [53.8%]) and *POR* (recipients: *1/*1, *n* = 21 [32.3%]; *1/*28, *n* = 32 [49.2%]; *28/*28, *n* = 12 [18.5%]; donors: *1/*1, *n* = 19 [29.2%]; *1/*28, *n* = 34 [52.3%]; *28/*28, *n* = 12 [18.5%]. Allele frequencies in our study were almost the same as the East Asian allele frequencies quoted from the 1000 Genomes Project (*CYP3A5*: *1/*1 = 8.2%; *1/*3 = 40.9%; *3/*3 = 50.9%, *POR*: *1/*1 = 39.2%; *1/*28 = 46.8%; *28/*28 = 14.0%) [26]. To evaluate the influence of combinations of recipient and donor *CYP3A5* genotypes, we divided the genotypes into two groups and the patients into four groups. One genotype group was a CYP3A5*1 group (CYP3A5*1/*1 and CYP3A5*1/*3), i.e., CYP3A5 expressors, and the other genotype group was a CYP3A5*3 group (CYP3A5*3/*3), i.e., CYP3A5 non-expressors. The patient groups were classified by the genotypes of both the recipient and donor. The four groups of patients were an R*1/D*1 group, in which both the recipient and the donor carried at least one CYP3A5*1 allele; an R*1/D*3 group, in which the recipient but not the donor carried a CYP3A5*1 allele; an R*3/D*1 group, in which the recipient had the CYP3A5*3/*3 genotype, while the donor carried a CYP3A5*1 allele; and an R*3/D*3 group, in which both the recipient and the donor had the CYP3A5*3/*3 genotype. Table 2 shows the classification of the patients by the combination of the recipient’s and donor’s *CYP3A5* polymorphisms. Of the total of 65 patient pairs, there were 21 pairs in the R*1/D*1 group, six pairs in the R*1/D*3 group, nine pairs in the R*3/D*1 group, and 29 pairs in the R*3/D*3 group. The *CYP3A5* and *POR* genotypes of the recipients are listed in Table 3A, and those of the donors are listed in Table 3B. There were eight recipients and nine donors in the F/*1 group, 19 recipients and 21 donors in the F/*28 group, 13 recipients and 10 donors in the D/*1 group, and 25 recipients and 25 donors in the D/*28 group.

### 2.3. Influence of the Recipient’s or Donor’s CYP3A5 Polymorphism on the TAC C/D Ratio during the First Month Following Liver Transplantation

**Figure 1:**
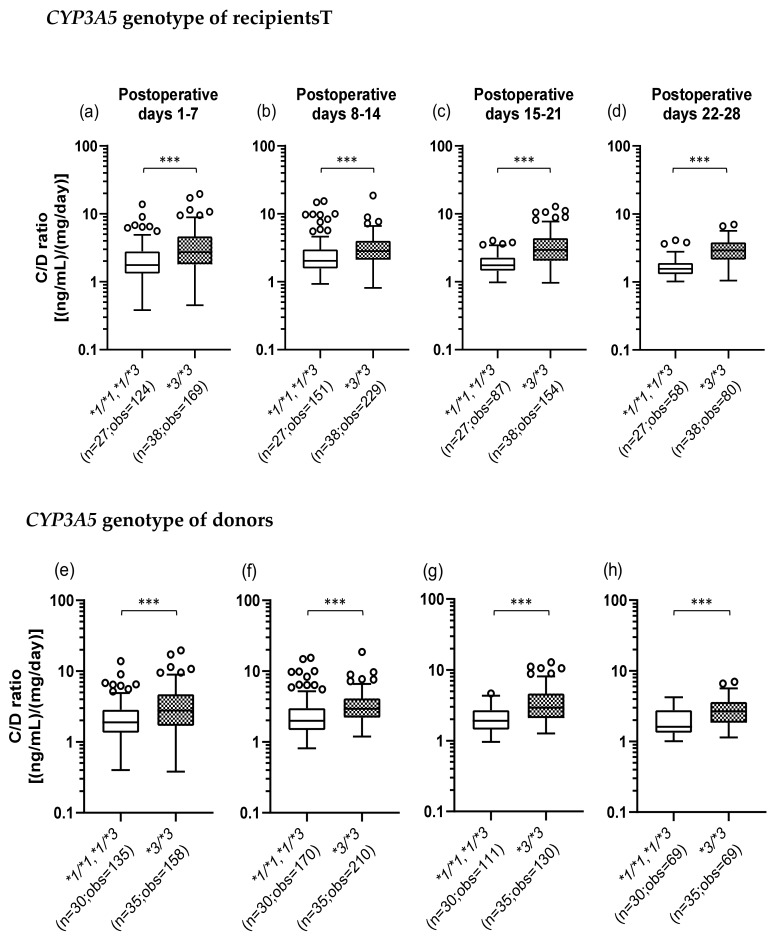
Influence of the recipient’s (**a**–**d**) or donor’s (**e**–**h**) *CYP3A5* polymorphism on the concentration/dose (C/D) ratio of tacrolimus on postoperative days 1–28 after living-donor liver transplantation. The C/D ratios of tacrolimus were compared on days 1–7 (a, e), 8–14 (b, f), 15–21 (c, g), and 22–28 (d, h) after transplantation for each *CYP3A5* genotype. The bar indicates the median tacrolimus C/D ratio for each group, and boxes represent the 25th and 75th percentiles of the data. The whiskers represent the lowest and highest values that fall within 1.5 times the interquartile range of the lower and upper quartiles, respectively. ***p < 0.001 between the groups. n, Number of patients; obs, number of observations i.e., number of tacrolimus troughs.

We investigated whether the CYP3A5*3 polymorphism affects the TAC C/D ratio. The number of TAC trough measurements decreased over time as some patients changed hospitals or for other reasons. Figure 1a–d shows that when the recipients did not express *CYP3A5* (*3/*3) (median C/D ratio [inter quartile range]: Pod 1–7, 2.73 [1.80–4.65]; Pod 8–14, 2.83 [2.10–4.00]; Pod 15–21, 2.94 [2.03–4.40]; Pod 22–28, 2.90 [2.14–3.83]), the TAC C/D ratios were significantly higher than those in the recipients expressing *CYP3A5* (*1/*1 and *1/*3) (median C/D ratio [inter quartile range]: Pod 1–7, 1.76 [1.32–2.79]; Pod 8–14, 2.03 [1.56–2.98]; Pod 15–21, 1.75 [1.45–2.26]; Pod 22–28, 1.55 [1.30–1.89]) (*p* < 0.001). Similarly, Figure 1e–h shows that the TAC C/D ratios were significantly higher when the donors had the CYP3A5*3/*3 genotype (median C/D ratio [inter quartile range]: Pod 1–7, 2.76 [1.68–4.70]; Pod 8–14, 2.93 [2.20–4.11]; Pod 15–21, 2.94 [2.09–4.65]; Pod 22–28, 2.67 [1.84–3.63]) compared to CYP3A5 expressors (median C/D ratio [inter quartile range]: Pod 1–7, 1.88 [1.35–2.85]; Pod 8–14, 1.99 [1.48–2.98]; Pod 15–21, 1.91 [1.44–2.70]; Pod 22–28, 1.61 [1.33–2.77]) (*p* < 0.001).

### 2.4. Influence of the Combination of the Recipient’s and Donor’s CYP3A5 Polymorphisms on the TAC C/D Ratio during the First Month Following Liver Transplantation

**Figure 2:**
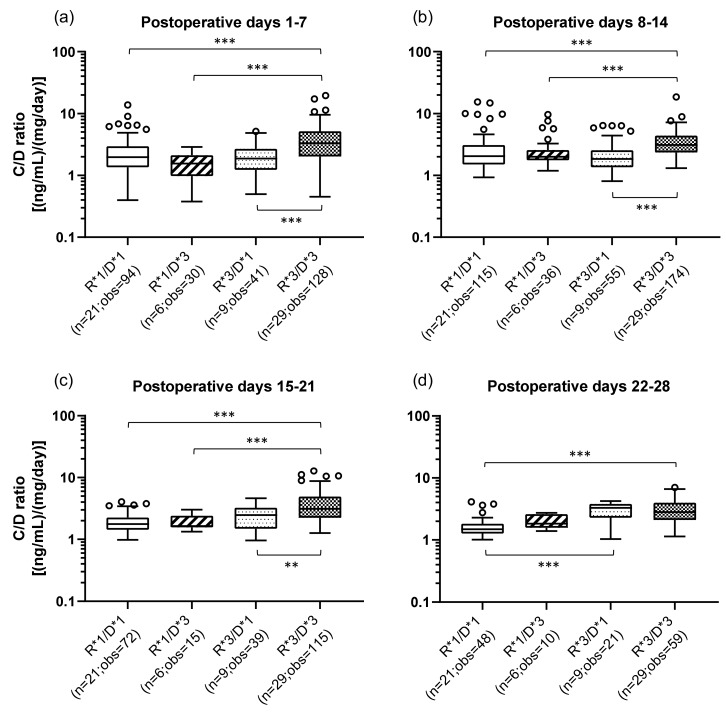
Influence of the combination of the recipient’s and donor’s *CYP3A5* genotypes on the C/D ratio of tacrolimus on postoperative days 1–28 after living-donor liver transplantation. The patient groups were divided into four groups by the recipient’s and donor’s *CYP3A5* polymorphism (R, recipient; D, donor; *1, CYP3A5*1/*1 and CYP3A5*1/*3; *3, CYP3A5*3/*3). The C/D ratios of tacrolimus were compared for days 1–7 (**a**), 8–14 (**b**), 15–21 (**c**), and 22–28 (**d**) after transplantation. The bar indicates the median tacrolimus C/D ratio for each group, and boxes represent the 25th and 75th percentiles of the data. The whiskers represent the lowest and highest values that fall within 1.5 times the interquartile range of the lower and upper quartiles, respectively. **p < 0.01 and ***p < 0.001 between groups. n, Number of patients; obs, number of observations i.e., number of tacrolimus troughs.

The effects of the combination of the recipient’s (small intestine) and donor’s (graft liver) *CYP3A5* polymorphisms on the TAC C/D ratios are shown in Figure 2. The classification method of the patients by *CYP3A5* polymorphisms and the number of the samples in each group are shown in Table 3. The R*3/D*3 group (median C/D ratio [inter quartile range]: Pod 1–7, 3.31 [2.03–5.16]; Pod 8–14, 3.10 [2.35–4.41]; Pod 15–21, 3.11 [2.23–4.90]; Pod 22–28, 2.83 [2.10–4.00]) showed a significantly higher C/D ratio than did the R*1/D*1 group (median C/D ratio [inter quartile range]: Pod 1–7, 1.96 [1.35–2.95]; Pod 8–14, 2.05 [1.50–3.10]; Pod 15–21, 1.77 [1.43–2.25]; Pod 22–28, 1.49 [1.27–1.82]) during all the observation periods (*p* < 0.001) (Figure 2a–d). In addition, compared with the R*1/D*3 group, the R*3/D*3 group had a significantly higher C/D ratio (*p* < 0.001) in the first to third weeks after transplantation. The same tendency was observed when comparing the R*3/D*3 group with the R*3/D*1 group (median C/D ratio [inter quartile range]: Pod 1–7, 1.88 [1.23–2.70]; Pod 8–14, 1.85 [1.35–2.57]; Pod 15–21, 2.48 [1.48–3.25]; Pod 22–28, 3.28 [2.29–3.81]) (second week: *p* < 0.001; second week: *p* < 0.001; third week: *p* = 0.002). Only in 4th week after transplantation, there was a significant difference between the R*1/D*1 and R*3/D*1 groups, with the latter showing a higher C/D ratio (*p* < 0.001).

### 2.5. Impact of Recipient’s or Donor’s POR*28 Genotype on the TAC C/D Ratio Requirement of a CYP3A5 Expressor or Non-Expressor during the First Month Following LIVER transplantation

**Figure 3:**
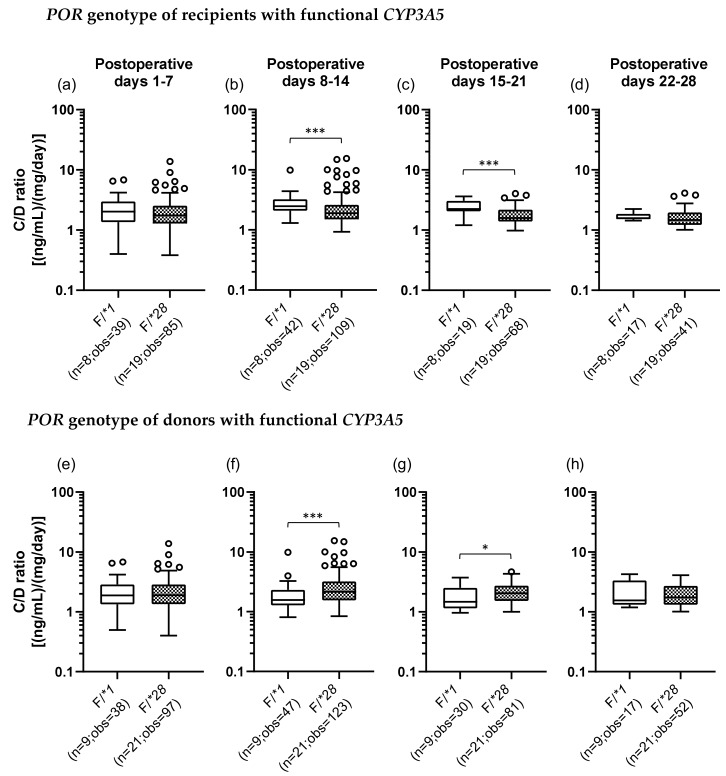
Impact of the POR*28 polymorphism in the recipients (**a**–**d**) and donors (**e**–**h**) with functional *CYP3A5* on the C/D ratio of tacrolimus on postoperative days 1–28 after living-donor liver transplantation. The C/D ratios of tacrolimus for days 1–7 (a, e), 8–14 (b, f), 15–21 (c, g), and 22–28 (d, h) after transplantation were compared by the *POR* polymorphism (F, functional; *1, POR*1/*1; and *28, POR*1/*28 and POR*28/*28). The bar indicates the median tacrolimus C/D ratio for each group, and boxes represent the 25th and 75th percentiles of the data. The whiskers represent the lowest and highest values that fall within 1.5 times the interquartile range of the lower and upper quartiles, respectively. *p < 0.05 and ***p < 0.001 between groups. n, Number of patients; obs, number of observations i.e., number of tacrolimus troughs.

We also examined the POR*28 polymorphism and classified *POR* genotypes into two groups, a POR*1 group, which was homozygous for the wild-type allele (POR*1/*1), and a POR*28 group, which carried at least one POR*28 allele (POR*1/*28 and POR*28/*28). In addition, we categorized each recipient and donor by the set of *CYP3A5* and *POR* polymorphisms. There were four groups as follows: the F/*1 group (F, functional), patients who carried a CYP3A5*1 allele and were homozygous for the POR*1 allele; the F/*28 group, patients who carried CYP3A5*1 and POR*28 alleles; the D/*1 group (D, defective), patients who did not carry CYP3A5*1 and POR*28 alleles; and the D/*28 group, patients who had the CYP3A5*3/*3 genotype and carried a POR*28 allele (Table 3). Figure 3 shows the results of univariate analysis between the F/*1 and F/*28 groups of recipients and donors. In the recipients with functional *CYP3A5*, the C/D ratio was significantly lower in the F/*28 group (median C/D ratio [inter quartile range]: Pod 1–7, 1.88 [1.23–2.70]; Pod 8–14, 1.85 [1.35–2.57]; Pod 15–21, 2.48 [1.48–3.25]; Pod 22–28, 3.28 [2.29–3.81]) than in the F/*1 group (median C/D ratio [inter quartile range]: Pod 1–7, 2.03 [1.35–2.96]; Pod 8–14, 2.47 [2.08–3.21]; Pod 15–21, 2.23 [2.05–3.06]; Pod 22–28, 1.57 [1.51–1.85]) in the second and third weeks after transplantation (*p* < 0.001) (Figure 3b,c). On the other hand, when the donors carried functional *CYP3A5*, the F/*28 group (median C/D ratio [inter quartile range]: Pod 1–7, 1.90 [1.34–2.85]; Pod 8–14, 2.15 [1.55–3.20]; Pod 15–21, 2.04 [1.53–2.72]; Pod 22–28, 1.74 [1.32–2.70]) showed a significantly higher C/D ratio than did the F/*1 group (median C/D ratio [inter quartile range]: Pod 1–7, 1.88 [1.34–2.86]; Pod 8–14, 1.57 [1.28–2.32]; Pod 15–21, 1.48 [1.15–2.51]; Pod 22–28, 1.55 [1.31–3.33]) in the second and third weeks after transplantation (second week: *p* < 0.001; third week: *p* = 0.015) (Figure 3f,g).

**Figure 4:**
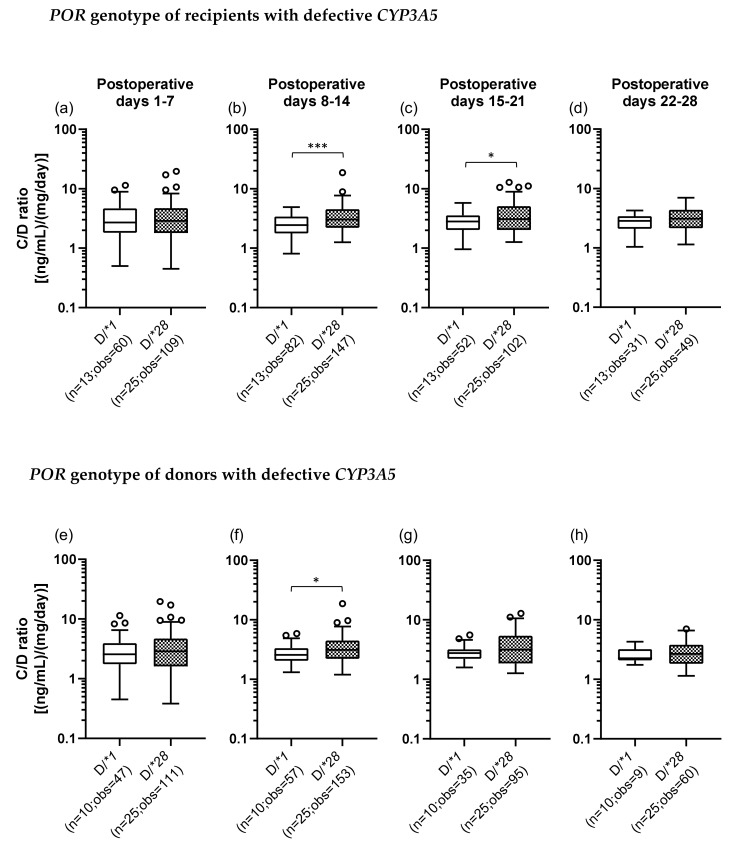
The impact of POR*28 polymorphism for recipient (**a**–**d**) or donor (**e**–**h**) with defective *CYP3A5* on the C/D ratio of tacrolimus for postoperative days 1–28 after living-donor liver transplantation. The C/D ratios of tacrolimus for 1–7 (a, e), 8–14 (b, f), 15–21 (c, g) and 22–28 (d, h) days after transplantation were compared by *POR* polymorphism (D, defective; *1, POR*1/*1; *28, POR*1/*28 and POR*28/*28). The bar indicates the median tacrolimus C/D ratio for each group and boxes represent the 25th and 75th percentiles of the data. The whiskers represent the lowest and highest values that fall within 1.5 times the interquartile range of the lower quartile and the upper quartile, respectively. *p < 0.05 and *** *p* < 0.001, between groups. n, number of patients; obs, number of observations i.e., number of tacrolimus troughs

The C/D ratios were also compared between the D/*1 and D/*28 groups of recipients and donors. In the recipients with defective *CYP3A5*, the C/D ratio was significantly higher in the D/*28 group (median C/D ratio [inter quartile range]: Pod 1–7, 2.88 [1.80–4.70]; Pod 8–14, 3.03 [2.20–4.53]; Pod 15–21, 3.06 [2.03–5.10]; Pod 22–28, 3.10 [2.15–4.38]) than in the D/*1 group (median C/D ratio [inter quartile range]: Pod 1–7, 2.55 [1.75–3.93]; Pod 8–14, 2.55 [2.04–3.33]; Pod 15–21, 2.75 [2.22–3.18]; Pod 22–28, 2.25 [2.08–3.20]) in the second and third weeks after transplantation (second week: *p* < 0.001; third week: *p* = 0.022) (Figure 4b,c). When the donors carried defective *CYP3A5*, the C/D ratio was significantly higher in the D/*28 group than in the D/*1 group in the second week after transplantation (*p* = 0.013) (Figure 4f).

### 2.6. Examination of Factors Affecting the TAC C/D Ratio Using Multiple Regression Analysis

To examine factors affecting the TAC C/D ratio during the first month after transplantation, multiple regression analyses were performed on five models, including all cases (Table 4, recipients with functional *CYP3A5* (Table 5), donors with functional *CYP3A5* (Table 6), recipients with defective *CYP3A5* (Table 7), and donors with defective *CYP3A5* (Table 8). Consequently, in the model of the multiple regression analysis of all cases, the recipient’s *CYP3A5* genotype was a significant variable contributing to the C/D ratios for all observation periods, whereas the donor’s *CYP3A5* genotype was a significant variable only in the third week after transplantation (Table 4).

## 3. Discussion

In this study, we investigated the effects of *CYP3A5* and *POR* polymorphisms in liver transplant recipients and donors on the pharmacokinetics of TAC during the first month after transplantation.

In liver transplantation, it is necessary to take into account the effects of *CYP3A5* polymorphisms in both the small intestine (recipient) and the liver (donor) on the TAC pharmacokinetics. In this study, univariate analyses showed that the TAC C/D ratio in the recipient was significantly higher, at least during the first month after transplantation, when the recipient or donor had the CYP3A5*3/*3 genotype than when both had CYP3A5*1/*1 and/or *1/*3 genotypes (Figure 1). These results were well comparable with previous findings showing that both the CYP3A5*3/*3 genotype of the small intestine and of the liver were significantly raised TAC C/D ratio in the liver transplant recipient [27,28,29]. Additionally, when the recipient’s and donor’s *CYP3A5* polymorphisms were examined in combination, the TAC C/D ratios in the R*1/D*3 and R*3/D*1 groups, in which either the recipient or the donor had functional *CYP3A5*, were lower in the first to third weeks after transplantation than that in the R*3/D*3 group, in which both the recipient and the donor had defective *CYP3A5*. These results suggested that CYP3A5 from both recipient’s small intestine and the donor-derived liver contributed to the TAC metabolism (Figure 2). Multiple regression analysis of the all patients showed that the recipient’s *CYP3A5* genotype was a significant variable contributing marker to the C/D ratio all four weeks after transplantation, however, the donor’s *CYP3A5* genotype was a significant variable contributing to the C/D ratio limited in the third week after transplantation (Table 4). Similarly, the contribution of the CYP3A5*3 polymorphism in the small intestine at an early after liver transplantation was previously reported [27,30]. In addition, previous reports have revealed that the contribution of CYP3A5 expressed in the donor-derived graft liver gradually increases with the recovery of liver function after transplantation [31]. Therefore, within at least one month after liver transplantation, CYP3A5 from the recipient’s small intestine contributed more to the TAC metabolism than CYP3A5 did from the donor-derived graft liver.

In this study, we also investigated, using multiple regression analysis, factors other than *CYP3A5*/*POR* gene polymorphisms that can affect the TAC C/D ratio. The results showed that a recipient’s sex, age, weight, and graft volume were often significant predictors of the TAC C/D ratio (Table 4, Table 5, Table 6 and Table 7). Thus, males consistently had lower C/D ratios when the recipient sex was included in the model as a significant predictor. CYP3A4 expression in the liver is higher in women [32,33,34], whereas no sex-related differences in the expression have been observed in the small intestine [35]. Our results suggest that males may have a higher TAC clearance in the small intestine than females at early stages after liver transplantation. However, few studies have examined the effects of sex differences on the CYP3A4 expression in the small intestine versus the liver, thus, further investigation is needed. Although age was positively correlated with the TAC C/D ratio, which was similar to the results of previous reports, suggesting that decreases in the liver volume and hepatic blood flow cause a decline in metabolic activity with age [36,37], the TAC C/D ratio negatively correlated with recipient’s weight and graft volume, consistent with general findings [38,39,40].

We also studied the effects of the POR*28 polymorphism on the TAC pharmacokinetics in liver transplant patients. When the recipients expressed functional *CYP3A5*, it was found that in the group in which recipients had the POR*28 polymorphism (F/*28), the TAC C/D ratio in the second and third weeks after transplantation was significantly lower than that in the group in which recipients did not have one (F/*1), suggesting that POR*28 polymorphism was a marker of the higher dosage of tacrolimus in patients carrying functional CYP3A5 (Figure 3a–d). Moreover, multiple regression analysis of the recipients with functional *CYP3A5* showed that the recipient’s *POR* genotype was a significant variable contributing to the C/D ratio in the third week after transplantation (Table 5). These results suggested that the POR*28 polymorphism decreased the TAC C/D ratio through an increase in the CYP3A5 activity. Similar results have been reported in studies targeting kidney transplant patients [21,22,24,41,42], heart transplant patients [43], and hematopoietic stem cell transplant patients [44]. However, this is the first report on liver transplant patients.

Although univariate analysis of the effect of the POR*28 polymorphism in donors with functional *CYP3A5* showed that the F/*28 group had a significantly higher TAC C/D ratio than did the F/*1 group in the second and third weeks after surgery (Figure 3f,g), multiple regression analysis showed that the POR*28 polymorphism in donors was not a significant predictor of the C/D ratio. These differences were due to the effects of confounding factors, and the contribution of the polymorphism was considered to be small, at least in the first month after transplantation (Table 6). Based on the previous findings [27,28,29,30,31]and the present results, the contribution of graft liver CYP3A5 on TAC metabolism was considered to be small compared to that in the native small intestine at early stage after liver transplantation. POR mediates the reduction of cytochrome P450 under aerobic conditions [45], but it is possible that POR did not function sufficiently early after transplantation, since the liver became ischemic during surgery. Therefore, the donor’s POR*28 polymorphism could not clearly affect TAC C/D ratio during the early period after surgery.

In addition, the effect of the POR*28 polymorphism on the TAC C/D ratio was examined in recipients with defective *CYP3A5*. The univariate analysis revealed that in the first month after transplantation, the TAC C/D ratio was significantly higher in the D/*28 group than in the D/*1 group at the second and third weeks after surgery, which was the opposite of the results obtained in the recipients with functional *CYP3A5* (Figure 4b,c). In multiple regression analysis, the recipient’s *POR* genotype was a significant predictor of the C/D ratio but limited in the second week after transplantation, which was not much different from the results of the univariate analysis (Table 7) and for which we have no reasonable explanation. Previous studies have shown that TAC is primarily metabolized by CYP3A4 when CYP3A5 is deficient [46], and the effect of the POR*28 polymorphism on CYP3A4 is controversial. For example, in vitro experiments using human liver microsomes showed no effect of the POR*28 polymorphism on CYP3A4 activity and expression [47]. On the other hand, in experiments using recombinant systems, with testosterone or midazolam used as a substrate for CYP3A4, the CYP3A4 activity was reduced by approximately 20%–40%, owing to the POR*28 polymorphism [48]. In vivo studies indicated that CYP3A4 activity was not affected by the POR*28 polymorphism [21,24,44]. However, there are reports showing that the activity was increased [22,49] and others suggesting that it was decreased [25]. Furthermore, a study that investigated the effect of the POR*28 polymorphism on the pharmacokinetics of cyclosporine, which is primarily metabolized by CYP3A4, suggested that the POR*28 polymorphism had little effect on CYP3A4 activity [23]. The results of the present study suggested that the POR*28 polymorphism tended to show a suppression of the CYP3A4 activity. Taken together, the influence of the POR*28 polymorphism on the CYP3A4 function should be further clarified, including the elucidation of the detailed biochemical mechanism.

There are some limitations in this study. First, the frequency of measurement of TAC trough concentrations differed among the patients because some patients were transferred to other hospitals at 2–3 weeks after transplantation, making it difficult to obtain measurement results. Therefore, the number of samples gradually decreased over time. Second, the drugs prescribed or the intake of food inhibiting or inducing CYP3A4 or CYP3A5 activity were not taken into consideration. Most patients used a combination of steroids, known to induce cytochrome P450 [50], and there were dose differences among the patients so that it cannot be ruled out that the TAC pharmacokinetics may have been affected. Further analysis with a larger sample size is required to assess the accuracy of the present results.

## 4. Materials and Methods

### 4.1. Patients

Initially, we included 109 Japanese recipients who underwent living-donor liver transplantation between July 2016 and January 2019 at the Kyushu University Hospital, as well as 109 donors, in this observational study. Among these subjects, 65 pairs of recipients and donors were included in the final study, who were over 18 years of age, signed written informed consent, and were treated with TAC as an immunosuppressant. Patients who died within 1 month after transplantation and those who switched from TAC to cyclosporine were excluded. We also excluded patients with cadaveric liver transplant because most liver transplantation cases in Japan (95%) are living-donor liver transplantation and the time from organ removal to transplantation in cadaveric liver transplantation is different to that in living-donor liver transplantation. This study was conducted in accordance with the Declaration of Helsinki and its later amendments and was approved by the Ethics Committee of the Kyushu University Graduate School and Faculty of Medicine (Approved number: 710-00, 8 March 2017).

For all patients, the following data were collected retrospectively: the sex of the recipient and donor, the age of the recipient and donor, the primary disease, body weight, graft size, preoperative laboratory test results (serum creatinine, blood urea nitrogen, liver function tests, and γ-glutamyl transpeptidase), and ABO blood group match.

### 4.2. DNA Extraction and Genotyping of the CYP3A5*3 and POR*28 Polymorphisms

Blood samples were collected from each recipient and donor, and DNA was extracted using the Wizard genomic DNA purification kit (Promega, Madison, WI, USA). All patients were genotyped for CYP3A5*3 (rs776746) and POR*28 (rs1057868) using real-time PCR TaqMan assays (StepOnePlus; Applied Biosystems, Waltham, MA, USA). DNA extraction and genotyping were performed in accordance with the manufacturers’ recommendations.

### 4.3. TAC Trough Concentration Measurement and Immunosuppression Protocol

TAC trough concentrations were measured as part of routine clinical care, and TAC doses were obtained for analysis from the electronic medical records. TAC (2–4 mg/day) was started within 3 days after surgery [51]. TAC dose was adjusted according to the clinical needs of the patient; the target whole-blood trough level was 10–12 ng/mL for the first month after transplantation. TAC trough level was measured almost every morning just before administration for the 1st week after transplantation. Thereafter, the measurement frequency was adjusted according to whether the trough level was stable or not. However, for most patients, there were some missing trough concentration data. The blood concentrations of TAC were measured using a chemiluminescent enzyme immunoassay (ARCHITECT; Abbott, Tokyo, Japan). The target trough concentrations were generally set to 10–12 ng/mL for the first month after transplantation. The C/D ratio was calculated by dividing the trough concentration by the previous day’s dose and used as an analysis index for TAC pharmacokinetics. Mycophenolate mofetil (2000–3000 mg/day) was initiated the morning after transplantation (Pod 1). Intravenous methylprednisolone (1000 mg) was administered immediately after portal vein reperfusion and hepatic artery reperfusion. This was tapered from 200 mg/day to 20 mg/day within 6 days and then tapered to 5 mg/day and occasionally stopped [51]. All patients received concomitant treatment with mycophenolate mofetil and a steroid, according to the posttransplant immunosuppressive program at the Kyushu University Hospital.

### 4.4. Statistical Analysis

Statistical analyses were carried out using the JMP version 14.2.0 software (SAS Institute, Inc., Cary, NC, USA). The Mann–Whitney *U*-test was used to assess the differences in the TAC C/D ratios. The Kruskal–Wallis test was used for comparisons among three or more groups. The chi-squared test was used to verify that the *CYP3A5* and *POR* genotype frequency distributions for our populations were consistent with the Hardy–Weinberg equilibrium (all *p* > 0.05). In addition to the *CYP3A5*/*POR* genotypes, the recipient’s sex/age/body weight and donor’s sex/age/graft weight were used as explanatory variables, and multiple regression analyses were performed using a stepwise method (variable reduction method). Box–Cox Y conversion was applied to normalize the residual between the predicted and measured values of the C/D ratio for each sample. The results were considered significant when *p*-values were lower than 0.05.

## 5. Conclusions

In conclusion, this study confirmed that the *CYP3A5* genotype of recipients, unlike that of donors, significantly contributed to the TAC C/D ratio, at least within the first month after liver transplantation. It was also found that the recipient’s POR*28 polymorphism affected the TAC pharmacokinetics when recipients had functional *CYP3A5*. These findings suggest that the combination of the *CYP3A5* and *POR* genotypes of recipients may be an indicator for early TAC dose adjustment after liver transplantation.

## Figures and Tables

**Table 1:** Characteristics of recipients and donors.

	Recipients (*n* = 65)	Donors (*n* = 65)
Preoperative data		
Sex (male/female)	28/37 (43.1/56.9)	43/22 (66.2/33.8)
Age (years)	59 (51–65)	39 (33–48)
Body weight (kg)	61.5 (54.0–67.9)	
Scr (mg/dL)	0.68 (0.52–0.90)	
BUN (mg/dL)	14 (10–19)	
eGFR (mL/min/1.73 m^2^)	78 (58–97)	
AST (U/L)	47 (31–74)	
ALT (U/L)	24 (17–38)	
γ-GTP (U/L)	36 (25–81)	
T-Bil (mg/dL)	2.5 (1.6–5.0)	
Alb (g/dL)	2.7 (2.4–3.0)	
Graft data		
GV (g)	490 (439–556)	
GRWR (%)	0.82 (0.69–0.91)	
Primary disease		
Alcoholic liver disease	16 (24.6)	
Primary biliary cholangitis	13 (20.0)	
Hepatitis C	10 (15.4)	
Hepatitis B	7 (10.8)	
Hepatocellular carcinoma	6 (9.2)	
Non-alcoholic steatohepatitis	5 (7.7)	
Autoimmune hepatitis	2 (3.1)	
Wilson’s disease	1 (1.5)	
Others	5 (7.7)	
ABO blood group match		
Identical	34 (52.3)	
Compatible	11 (16.9)	
Incompatible	20 (30.8)	

Continuous data are presented as the median (25th to 75th quartiles); categorical data are presented as numbers (%). Scr, serum creatinine; BUN, blood urea nitrogen; eGFR, estimated glomerular filtration rate; AST, aspartate aminotransferase; ALT, alanine aminotransferase; γ-GTP, gamma-glutamyl transpeptidase; T-Bil, total bilirubin; Alb, serum albumin; GV, graft volume; GRWR, graft–recipient weight ratio.

**Table 2:** Classification of the patients by the combination of the recipient’s and donor’s *CYP3A5* polymorphisms.

Category	n (%)	Recipient’s *CYP3A5* Genotype	Donor’s *CYP3A5* Genotype
R*1/D*1	21 (32.3)	*1/*1, *1/*3	*1/*1, *1/*3
R*1/D*3	6 (9.2)	*1/*1, *1/*3	*3/*3
R*3/D*1	9 (13.8)	*3/*3	*1/*1, *1/*3
R*3/D*3	29 (44.6)	*3/*3	*3/*3

R, recipient; D, donor; *1, CYP3A5*1/*1 or *1/*3; *3, CYP3A5*3/*3.

**Table 3:** Demographics of the recipients and donors by the combination of *CYP3A5* and *POR* genotypes.

**A)** Categorization of the recipients by *CYP3A5* and *POR* genotypes			
Category	n (%)	Recipient’s *CYP3A5* Genotype	Recipient’s *POR* Genotype
F/*1	8 (12.3)	*1/*1, *1/*3	*1/*1
F/*28	19 (29.2)	*1/*1, *1/*3	*1/*28, *28/*28
D/*1	13 (20.0)	*3/*3	*1/*1
D/*28	25 (38.5)	*3/*3	*1/*28, *28/*28
**B)** **Categorization of the donors by *CYP3A5* and *POR* genotypes**			
**Category**	**n (%)**	**Donor’s *CYP3A5* Genotype**	**Donor’s *POR* Genotype**
F/*1	9 (13.8)	*1/*1, *1/*3	*1/*1
F/*28	21 (32.3)	*1/*1, *1/*3	*1/*28, *28/*28
D/*1	10 (15.4)	*3/*3	*1/*1
D/*28	25 (38.5)	*3/*3	*1/*28, *28/*28

F, functional *CYP3A5*; D, defective *CYP3A5*; *1, POR*1/*1; *28, POR*1/*28 or *28/*28.

**Table 4:** Multiple regression analysis of all patients; (B) Multiple regression analysis of cases in which recipients had functional *CYP3A5;* (**C**).

All Patients (65)	Pod 1–7		Pod 8–14		Pod 15–21		Pod 22–28	
	β	*p*	β	*p*	Β		*p*	*p*
Recipient sex (male (28) vs. female (37))	−0.184	0.002	−	−	−	−	−	−
Recipient age (years)	0.156	0.002	0.095	0.018	0.100	0.043	−	
Recipient BW (kg)	−0.262	<0.001	−0.185	<0.001	−0.138	0.008	−	−
Donor sex (male (43) vs. female (22))	−	−	−	−	−	−	−	−
Donor age (years)	−	−	−	−	−	−	−	−
Graft volume (g)	−0.157	0.003	−0.520	<0.001	−0.383	<0.001	−0.274	<0.001
Recipient *CYP3A5* (f (27) vs. d (38))	−0.389	<0.001	−0.345	<0.001	−0.417	<0.001	−0.627	<0.001
Donor *CYP3A5* (f (30) vs. d (35))	−	−	−	−	−0.175	0.004	−	−
Recipient *POR* (*1 (21) vs. *28 (44))	−	−	−	−	−	−	−	−
Donor *POR* (*1 (19) vs. *28 (46))	−	−	−	−	−	−	−	−

The corresponding places in the table were showed “–”, for the explanatory variables excluded from the regression equation by the stepwise method. Pod, postoperative days; β, standard partial regression coefficient; f, functional *CYP3A5* (*1/*1 or *1/*3); d, defective *CYP3A5* (*3/*3); *1, POR*1/*1; *28, POR*1/*28 or *28/*28.

**Table 5:** Multiple regression analysis of cases in which recipients had functional *CYP3A5.*

Recipient/ Functional *CYP3A5* (27)	Pod 1–7		Pod 8–14		Pod 15–21		Pod 22–28	
	β	*p*	β	*p*	Β	*p*	β	*p*
Recipient sex (male (11) vs. female (16))	−0.304	0.001	−0.340	<0.001	−	−	−0.440	0.001
Recipient age (years)	0.184	0.027	−	−	0.641	<0.001	0.941	<0.001
Recipient BW (kg)	−0.262	<0.001	−	−	−0.352	<0.001	−0.360	0.008
Donor sex (male (19) vs. female (8))	−	−	−	−	−	−	0.168	0.144
Donor age (years)	−	−	−	−	0.545	<0.001	1.162	<0.001
Graft volume (g)	−0.176	0.022	−0.545	<0.001	−0.737	<0.001	−0.872	<0.001
Donor *CYP3A5* (f (21) vs. d (6))	−	−	−	−	−0.394	<0.001	−0.579	<0.001
Recipient *POR* (*1 (8) vs. *28 (19))	−	−	−	−	0.390	<0.001	−	−
Donor *POR* (*1 (9) vs. *28 (18))	−	−	−	−	−	−	−	−

The corresponding places in the table were showed “–”, for the explanatory variables excluded from the regression equation by the stepwise method. Pod, postoperative days; β, standard partial regression coefficient; f, functional *CYP3A5* (*1/*1 or *1/*3); d, defective *CYP3A5* (*3/*3); *1, POR*1/*1; *28, POR*1/*28 or *28/*28.

**Table 6:** Multiple regression analysis of cases in which donors had functional *CYP3A5.*

Donor/ Functional *CYP3A5* (30)	Pod 1–7		Pod 8–14		Pod 15–21		Pod 22–28	
	β	*p*	β	*p*	β	*p*	β	*p*
Recipient sex (male (13) vs. female (17))	−	−	−	−	−	−	−	−
Recipient age (years)	−	−	−	−	0.312	<0.001	0.187	0.074
Recipient BW (kg)	−0.283	<0.001	−	−	−	−	−	−
Donor sex (male (21) vs. female (9))	−	−	−	−	−	−	−	−
Donor age (years)					0.310	<0.001	−	−
Graft volume (g)	−0.178	0.032	−0.617	<0.001	−0.730	<0.001	−0.301	0.006
Recipient *CYP3A5* (f (21) vs. d (9))	−	−	−	−	−0.436	<0.001	−0.688	<0.001
Recipient *POR* (*1 (11) vs. *28 (19))	−	−	−	−	−	−	−	−
Donor *POR* (*1 (9) vs. *28 (21))	−	−	−	−	−	−	−	−

The corresponding places in the table were showed “–”, for the explanatory variables excluded from the regression equation by the stepwise method. Pod, postoperative days; β, standard partial regression coefficient; f, functional *CYP3A5* (*1/*1 or *1/*3); d, defective *CYP3A5* (*3/*3); *1, POR*1/*1; *28, POR*1/*28 or *28/*28.

**Table 7:** Multiple regression analysis of cases in which recipients had defective *CYP3A5.*

Recipient/ Defective *CYP3A5* (38)	Pod 1–7		Pod 8–14		Pod 15–21		Pod 22–28	
	β	*p*	β	*p*	β	*p*	β	*p*
Recipient sex (male (17) vs. female (21))	−0.198	0.009	−0.205	<0.001	−	−	−	−
Recipient age (years)	−	−	0.214	<0.001	−	−	−	−
Recipient BW (kg)	−	−	−0.332	<0.001	−0.221	0.004	−0.288	0.011
Donor sex (male (24) vs. female (14))	−	−	−	−	−	−	−	−
Donor age (years)	−	−	−	−	−	−	−0.258	0.022
Graft volume (g)	−0.182	0.024	−0.434	<0.001	−0.443	<0.001	−	−
Donor *CYP3A5* (f (9) vs. d (29))	−0.256	<0.001	−	−	−	−	−	−
Recipient *POR* (*1 (13) vs. *28 (25))	−	−	−0.168	0.002	−	−	−	−
Donor *POR* (*1 (10) vs. *28 (28))	−	−	−	−	−	−	−	−

The corresponding places in the table were showed “–”, for the explanatory variables excluded from the regression equation by the stepwise method. Pod, postoperative days; β, standard partial regression coefficient; f, functional *CYP3A5* (*1/*1 or *1/*3); d, defective *CYP3A5* (*3/*3); *1, POR*1/*1; *28, POR*1/*28 or *28/*28.

**Table 8:** Multiple regression analysis of cases in which donors had defective *CYP3A5.*

Donor/ Defective *CYP3A5* (35)	Pod 1–7		Pod 8–14		Pod 15–21		Pod 22–28	
	β	*p*	β	*p*	β	*p*	β	*p*
Recipient sex (male (15) vs. female (20))	−0.322	<0.001	−	−	−	−	−	−
Recipient age (years)	−	−	0.305	<0.001	0.138	<0.001	−	−
Recipient BW (kg)	−	−	−0.664	<0.001	−0.564	<0.001	−0.495	<0.001
Donor sex (male (22) vs. female (13))	−	−	0.265	<0.001	−	−	−	−
Donor age (years)	−	−	−	−	−	−	−	−
Graft volume (g)	−0.133	0.049	−	−	−	−	−	−
Recipient *CYP3A5* (f (6) vs. d (29))	−0.413	<0.001	−	−	−0.289	<0.001	−0.309	0.004
Recipient *POR* (*1 (10) vs. *28 (25))	−	−	−0.050	0.352	−0.098	0.152	−0.155	0.133
Donor *POR* (*1 (10) vs. *28 (25))	−	−	−	−	−	−	−	−

The corresponding places in the table were showed “–”, for the explanatory variables excluded from the regression equation by the stepwise method. Pod, postoperative days; β, standard partial regression coefficient; f, functional *CYP3A5* (*1/*1 or *1/*3); d, defective *CYP3A5* (*3/*3); *1, POR*1/*1; *28, POR*1/*28 or *28/*28.
